# Effectiveness of VISIA system in evaluating the severity of rosacea

**DOI:** 10.1111/srt.13194

**Published:** 2022-07-11

**Authors:** Yu Pan, Kaiyu Jia, Sihan Yan, Xian Jiang

**Affiliations:** ^1^ Department of Dermatology West China Hospital Sichuan University Chengdu China; ^2^ Laboratory of Dermatology, Clinical Institute of Inflammation and Immunology, Frontiers Science Center for Disease‐related Molecular Network West China Hospital Sichuan University Chengdu China

**Keywords:** erythema, noninvasive measurement, rosacea, VISIA

## Abstract

**Background:**

Rosacea is a facial chronic inflammatory skin disease with almost 5.5% prevalence. Although there are various scales of rosacea, they are objective and discordant among different dermatologists. Noninvasive objective measurements such as VISIA system might play essential roles in the diagnosis and evaluation of rosacea. Here, we intended to reveal the effectiveness of VISIA system in rosacea.

**Materials and methods:**

A number of 563 participants diagnosed with facial rosacea were enrolled in study. They all received both full‐face image‐shoot by VISIA system with quantitative analysis software and physician's assessment via five different scales, including investigator global assessment (IGA), clinician erythema assessment (CEA), numerical score, the National Rosacea Society (NRS) grading system and telangiectasis.

**Results:**

Absolute score and percentile of red area had significant correlations with IGA and CEA, whereas red area had no significant correlation with numerical score, NRS and telangiectasis. Red area in erythematotelangiectatic rosacea patients demonstrated the highest correlation with IGA and CEA, especially in those aged between 51 and 60. Besides red area, pigmentation parameters in VISIA system (brown spot) also showed significant correlation with IGA and CEA.

**Conclusion:**

VISIA system might be an effective measurement in the assessment of rosacea severity.

## 1 INTRODUCTION

Rosacea is a chronic inflammatory skin disorder that mainly affects the central face, characterized by flushing, erythema, papule, pustule and telangiectasia.^1^ The global prevalence of rosacea is about 5.46% based on published data. However, the prevalence in populations with skin of color might be underreported because of the difficulty of diagnosis.^2^ There are originally four subtypes of rosacea, including erythematotelangiectatic rosacea (ETR), papulopustular rosacea (PPR), phymatous rosacea (PhR) and ocular rosacea (OR).^3^ Recently, the classification has been updated from subtype‐based to phenotype‐based considering the growing knowledge of rosacea pathophysiology.^4^, ^5^ However, considering the diversity and complexity of clinical manifestations in rosacea patients, the various classification systems and the nonspecific histopathology of rosacea, it is challenging to standardize and quantify the measurements of rosacea.^6^ Although there are numerous scales of rosacea, those scales are subjective and not reliable.

A noninvasive measurement technique provides a novel method for diagnosis and evaluation of skin diseases without damage. Noninvasive tools have been applied for the diagnosis and estimation of different skin disorders, such as scars,^7^ vitiligo,^8^ melanoma,^9^ acne,^10^ psoriasis,^11^ eczema^12^ and port wine stain.^13^ The emerging imaging technology, serving as one of these important noninvasive methods, is beneficial for dermatologists to diagnose and evaluate patients precisely and rapidly. VISIA system (Canfield Scientific, Parsippany, NJ, USA) is a common noninvasive imaging system, available to capture high‐resolution facial images.^9^, ^14^ By using quantitative analysis software (RBX; Canfield Scientific), the VISIA system separates red and brown channels and enables a better visualization of erythema and telangiectasia.^15^

In this article, we compared VISIA parameters with physician's subjective assessments and analyzed their correlations to clarify the effectiveness of VISIA system in evaluating rosacea.

## 2 MATERIALS AND METHODS

### 2.1 Study design and participants

This trial (ChiCTR2200058297) was a retrospective cross‐sectional study with the approval of ethics committees in West China Hospital and conducted in accordance with the Declaration of Helsinki. During January 1, 2018 to May 1, 2021, patients diagnosed with rosacea, classified according to the 2002 National Rosacea Society (NRS) classification system,^3^ with written informed consent prior to participation were eligible. Participants diagnosed with OR without facial lesion, pregnant or breastfeeding, combined with severe mental disorders or systemic diseases (including respiratory, digestive, circulatory, skeletal and muscular, and immune system diseases) were excluded.

### 2.2 Measurements

**FIGURE 1:**
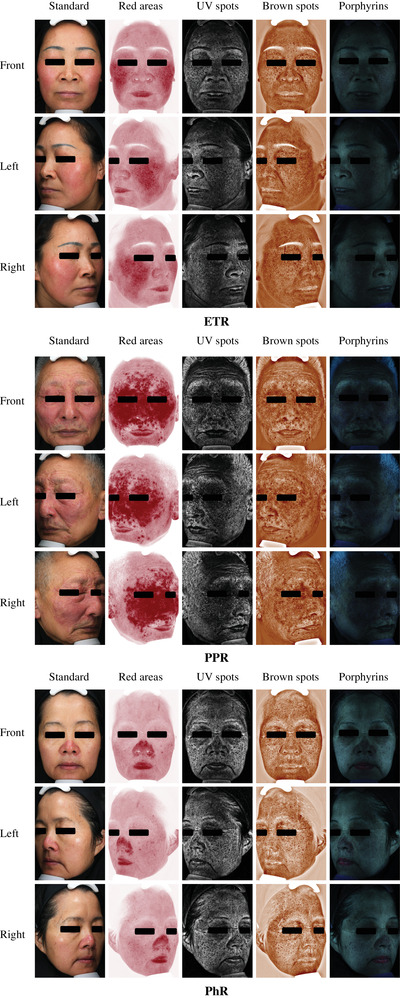
VISIA images of rosacea patients in different subtypes: erythematotelangiectatic rosacea (ETR), papulopustular rosacea (PPR), phymatous rosacea (PhR)

All participants received front, left and right sides of full‐face images by VISIA system (Canfield Scientific, Parsippany, NJ, USA), consisting of standard, cross‐polarized and ultraviolet (UV) photography, respectively. Through analysis by RBX technology,^15^ feature count, absolute score and percentile of spot, wrinkle, texture, pore, UV spot, red area and porphyrin were obtained (Figure 1). Before shooting, patients had cleaned their face and rested in a controlled dark condition (20–24°C, 48%–50% relative humidity) for 30 min.

### 2.3 Physician's assessments (PSA)

One certified dermatologist assessed patients’ standard images according to five scales, respectively, including investigator global assessment (IGA),^16^ clinician erythema assessment (CEA),^17^ numerical score,^18^ the NRS grading system and telangiectasis.^6^

### 2.4 Statistical analysis

Spearman correlation analyses between VISIA parameters and physician's assessment (PSA) were determined by Stata 16.0 software.

## 3 RESULTS

### 3.1 Study flow and demographic characteristics

**FIGURE 2:**
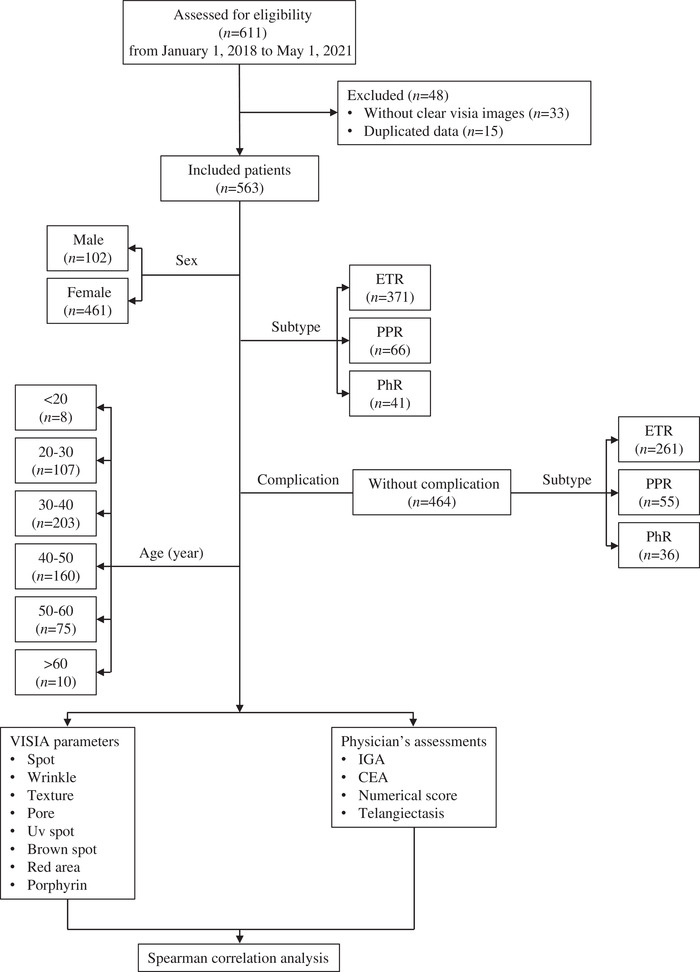
Study flow physician's assessment (PSA), investigator global assessment (IGA), clinician erythema assessment (CEA), National Rosacea Society (NRS)

A number of 611 subjects were screened, and 563 of them entered the study (Figure 2). The mean age was 39‐year old, with 102 male patients and 461 female rosacea patients, respectively (Table 1). A number of 85 (15.1%) of them were diagnosed rosacea with complications, such as nevus, acne, melasma, vitiligo and freckle. Percentages of ETR, PPR and PhR were 56.31%, 11.72% and 7.28% separately. A number of 139 patients (24.69%) could not be easily classified with only one subtype because they encompassed a multitude combination of signs and symptoms.

**TABLE 1:** Demographic characteristics of rosacea patients

		Subtype							Complication		
Sex	Age	ETR	PPR	PhR	ETR + PPR	ETR + PhR	PPR + PhR	PPR + ETR + PhR	Without	With	Total
Male	≤20	0 (0)	1 (0.18)	0 (0)	0 (0)	0 (0)	0 (0)	0 (0)	1 (0.18)	0 (0)	1 (0.18)
	21–30	1 (0.18)	5 (0.89)	4 (0.71)	0 (0)	8 (1.42)	3 (0.53)	1 (0.18)	15 (2.66)	7 (1.24)	22 (3.91)
	31–40	8 (1.42)	1 (0.18)	22 (3.91)	2 (0.36)	1 (0.18)	0 (0)	0 (0)	29 (5.15)	5 (0.89)	34 (6.04)
	41–50	1 (0.18)	1 (0.18)	1 (0.18)	1 (0.18)	15 (2.66)	0 (0)	1 (0.18)	19 (3.37)	1 (0.18)	20 (3.55)
	51–60	5 (0.89)	0 (0)	2 (0.36)	0 (0)	7 (1.24)	0 (0)	2 (0.36)	16 (2.84)	0 (0)	16 (2.84)
	≥61	2 (0.36)	1 (0.18)	0 (0)	0 (0)	6 (1.07)	0 (0)	0 (0)	8 (1.42)	1 (0.18)	9 (1.60)
	Total	17 (3.02)	9 (1.60)	29 (5.15)	3 (0.53)	37 (6.57)	3 (0.53)	4 (0.71)	88 (15.63)	14 (2.49)	102 (18.12)
Female	≤20	4 (0.71)	0 (0)	1 (0.18)	2 (0.36)	0 (0)	0 (0)	0 (0)	5 (0.89)	2 (0.36)	7 (1.24)
	21–30	54 (9.59)	13 (2.31)	1 (0.18)	16 (2.84)	0 (0)	1 (0.18)	0 (0)	79 (14.03)	6 (1.07)	85 (15.10)
	31–40	95 (16.87)	17 (3.02)	7 (1.24)	42 (7.46)	8 (1.42)	0 (0)	0 (0)	141 (25.04)	28 (4.97)	169 (30.02)
	41–50	96 (17.05)	27 (4.80)	2 (0.36)	8 (1.42)	4 (0.71)	0 (0)	3 (0.53)	106 (18.83)	34 (6.04)	140 (24.87)
	51–60	50 (8.88)	0 (0)	1 (0.18)	4 (0.71)	4 (0.71)	0 (0)	0 (0)	45 (7.99)	14 (2.49)	59 (10.48)
	≥61	1 (0.18)	0 (0)	0 (0)	0 (0)	0 (0)	0 (0)	0 (0)	0 (0)	1 (0.18)	1 (0.18)
	Total	300 (53.29)	57 (10.12)	12 (2.13)	72 (12.79)	16 (2.84)	1 (0.18)	3 (0.53)	376 (66.79)	85 (15.10)	461 (81.88)
Total		317 (56.31)	66 (11.72)	41 (7.28)	75 (13.32)	53 (9.41)	4 (0.71)	7 (1.24)	464	99	563 (100)

Abbreviations: ETR, erythematotelangiectatic rosacea; PhR, phymatous rosacea; PPR, papulopustular rosacea.

### 3.2 Correlations between PSA and red area

All three indexes (feature count, absolute score and percentile) of red area in the left and right faces had significant correlations with IGA and CEA, whereas our study showed no significant correlation between PSA and feature count of red area in the front face (Table 2). However, red area showed no significant correlation with numerical score, NRS and telangiectasis.

**TABLE 2:** Correlation analysis between physician's assessment (PSA) and red area

	Red area								
	Feature count			Absolute score			Percentile		
PSA	Front	Left	Right	Front	Left	Right	Front	Left	Right
IGA	0.1150*	0.3919*	0.4208*	0.4758*	0.4507*	0.4344*	−0.3883*	−0.4862*	−0.4646*
CEA	0.0696	0.4237*	0.4444*	0.3790*	0.4580*	0.4125*	−0.3389*	−0.4711*	−0.4400*
Numerical score	0.0714	0.0392	0.0651	0.1264*	0.1754*	0.1876*	−0.0757	−0.2154*	−0.2078*
NRS	0.0857*	0.0612	0.0833*	0.1375*	0.1736*	0.1882*	−0.0825	−0.2072*	−0.2121*
Telangiectasis	0.0556	0.2403*	0.2699*	0.3468*	0.2539*	0.2364*	−0.2879*	−0.2248*	−0.1815*

Abbreviations: CEA, clinician erythema assessment; IGA, investigator global assessment; NRS, National Rosacea Society.

* *p* < 0.05.

*Note*: *𝛂* = 0.05;.

We further investigated the differences of correlations among various rosacea subtypes. As results demonstrated, the absolute score and percentile of red area in the left and right sides had significant correlations with IGA and CEA in ETR and PPR patients, with higher *r* value in ETR (Table 3). Although there was no significant correlation between red area and IGA (or CEA), feature count of red area in the front face displayed significant correlation with numerical score, NRS and telangiectasis.

**TABLE 3:** Correlation analysis between physician's assessment (PSA) and red area in different subtypes

		Red area								
		Feature count			Absolute score			Percentile		
Subtype	PSA	Front	Left	Right	Front	Left	Right	Front	Left	Right
ETR	IGA	0.0074	0.3000*	0.3420*	0.4723*	0.3734*	0.3814*	−0.3800*	−0.4632*	−0.4701*
	CEA	−0.0282	0.2726*	0.2960*	0.3925*	0.3642*	0.3257*	−0.3044*	−0.3901*	−0.3727*
	Numerical score	−0.0787	−0.0227	0.0093	−0.0027	0.0639	0.103	0.0436	−0.059	−0.0674
	NRS	0.0558	−0.0227	−0.0109	0.0328	0.0639	0.0689	−0.0313	−0.059	−0.0693
	Telangiectasis	−0.0311	0.1302*	0.1644*	0.3833*	0.2091*	0.1990*	−0.2351*	−0.2389*	−0.1914*
PPR	IGA	0.0322	0.223	0.161	0.4434*	0.3398*	0.3279*	−0.4326*	−0.3348*	−0.4080*
	CEA	−0.114	0.4826*	0.4273*	0.192	0.4746*	0.4392*	−0.3139*	−0.4535*	−0.4680*
	Numerical score	0.0711	0.072	0.0685	0.119	0.193	0.2600*	−0.215	−0.3223*	−0.3524*
	NRS	−0.022	0.0901	0.133	0.0337	0.222	0.2970*	−0.161	−0.235	−0.3791*
	Telangiectasis	−0.057	0.4261*	0.4490*	0.179	0.3122*	0.2550*	−0.231	−0.102	0.0887
PhR	IGA	0.0476	0.0836	0.234	0.261	0.172	0.212	−0.16	−0.205	−0.083
	CEA	−0.0893	0.108	0.229	0.251	0.199	0.208	−0.116	−0.241	−0.186
	Numerical score	0.4213*	0.191	0.259	0.0759	0.185	0.247	0.0697	−0.0568	−0.0228
	NRS	0.3426*	0.128	0.183	0.23	0.0733	0.14	0.0616	0.0108	0.0409
	Telangiectasis	0.3401*	0.142	0.15	0.6089*	0.174	0.135	−0.4298*	−0.0951	−0.0079

Abbreviations: CEA, clinician erythema assessment; ETR, erythematotelangiectatic rosacea; IGA, investigator global assessment; NRS, National Rosacea Society; PhR, phymatous rosacea; PPR, papulopustular rosacea.

* *p* < 0.05.

*Note*: *𝛂* = 0.05.

Except the absolute score of red area in the front face, the absolute score in the left and right side had higher *r* value in male rosacea patients than in female rosacea patients (Table 4). Similarly, the feature count and percentile of red area in male patients showed higher correlation compared with those in female patients.

**TABLE 4:** Correlation analysis between physician's assessment (PSA) and red area in different sexes

		Red area								
		Feature count			Absolute score			Percentile		
Sex	PSA	Front	Left	Right	Front	Left	Right	Front	Left	Right
Male	IGA	0.3586*	0.4946*	0.5264*	0.4042*	0.5231*	0.4909*	−0.4756*	−0.5753*	−0.5134*
	CEA	0.2369*	0.6060*	0.6110*	0.3132*	0.6692*	0.5921*	−0.4446*	−0.6278*	−0.5681*
	Numerical score	0.154	0.138	0.157	0.0654	0.2464*	0.2254*	0.0314	−0.3185*	−0.2831*
	NRS	0.121	0.158	0.182	0.085	0.2014*	0.2157*	0.0233	−0.2820*	−0.2691*
	Telangiectasis	0.2164*	0.4594*	0.4494*	0.4151*	0.4700*	0.4294*	−0.3554*	−0.3693*	−0.3539*
Female	IGA	0.0537	0.3527*	0.3798*	0.5008*	0.4265*	0.4160*	−0.3747*	−0.4495*	−0.4392*
	CEA	0.0296	0.3501*	0.3806*	0.4088*	0.3990*	0.3581*	−0.3225*	−0.4015*	−0.3770*
	Numerical score	0.0446	0.0162	0.0326	0.1379*	0.1671*	0.1811*	−0.0978*	−0.2044*	−0.1959*
	NRS	0.0692	0.0388	0.0482	0.1496*	0.1741*	0.1866*	−0.1054*	−0.1991*	−0.2049*
	Telangiectasis	0.0224	0.1960*	0.2296*	0.3502*	0.2103*	0.1969*	−0.2790*	−0.1788*	−0.1276*

Abbreviations: CEA, clinician erythema assessment; IGA, investigator global assessment; NRS, National Rosacea Society.

* *p* < 0.05.

*Note*: *𝛂* = 0.05.

In order to study correlations among different age groups, we divided all patients into six groups (Table 5). Participants between 51 and 60 years demonstrated the highest *r* value between red area and IGA (or CEA), whereas there seemed no significant correlation between red area and PSA in rosacea patients aged lower than 20 or older than 60.

**TABLE 5:** Correlation analysis between physician's assessment (PSA) and red area in different ages

		Red area								
		Feature count			Absolute score			Percentile		
Age	PSA	Front	Left	Right	Front	Left	Right	Front	Left	Right
≤20	IGA	0.154	0.063	−0.126	0.8487*	−0.567	−0.378	−0.53	0.452	0.127
	CEA	−0.327	−0.055	−0.344	0.218	−0.7698*	−0.536	−0.25	0.69	−0.0138
	Numerical score	−0.0273	−0.355	−0.0273	0.3	−0.218	0.109	−0.188	0.335	0.165
	NRS	0	0.247	0.247	0.378	−0.0825	0.0825	−0.144	0.0845	−0.166
	Telangiectasis	−0.327	−0.7638*	−0.7638*	0.218	−0.7638*	−0.655	−0.25	0.7826*	0.494
21–30	IGA	−0.0335	0.2168*	0.2040*	0.4622*	0.3971*	0.3715*	−0.3507*	−0.5815*	−0.4922*
	CEA	−0.0448	0.2683*	0.3010*	0.3781*	0.3644*	0.3742*	−0.2491*	−0.4526*	−0.4276*
	Numerical score	−0.0755	−0.0637	−0.0785	0.1922*	0.106	0.0978	−0.168	−0.2783*	−0.2202*
	NRS	−0.08	−0.0516	−0.0732	0.1979*	0.0985	0.0781	−0.153	−0.2490*	−0.189
	Telangiectasis	−0.0438	0.2127*	0.2324*	0.2315*	0.2534*	0.2684*	−0.3824*	−0.115	−0.136
31–40	IGA	0.2471*	0.4514*	0.4767*	0.5053*	0.4554*	0.4389*	−0.3651*	−0.4752*	−0.4378*
	CEA	0.2228*	0.4259*	0.4374*	0.4671*	0.4461*	0.3769*	−0.3617*	−0.5108*	−0.4290*
	Numerical score	0.2398*	0.1776*	0.2173*	0.1388*	0.2798*	0.2944*	−0.119	−0.2869*	−0.2789*
	NRS	0.2647*	0.1899*	0.2163*	0.1626*	0.2868*	0.2988*	−0.1408*	−0.2925*	−0.2915*
	Telangiectasis	0.2661*	0.107	0.111	0.3806*	0.111	0.0572	−0.1912*	−0.2151*	−0.0852
41–50	IGA	0.0752	0.4002*	0.4522*	0.3242*	0.4168*	0.3996*	−0.3135*	−0.3815*	−0.4403*
	CEA	−0.0049	0.4446*	0.4918*	0.2207*	0.4654*	0.4097*	−0.2783*	−0.3591*	−0.3769*
	Numerical score	−0.0373	−0.0391	−0.003	0.0923	0.149	0.1700*	0.0251	−0.107	−0.142
	NRS	−0.0362	−0.0401	0.0102	0.0721	0.111	0.1571*	0.0375	−0.0815	−0.145
	Telangiectasis	−0.121	0.2893*	0.3365*	0.2678*	0.3015*	0.2876*	−0.2411*	−0.2635*	−0.2239*
51–60	IGA	0.0152	0.5045*	0.4888*	0.5738*	0.6325*	0.6038*	−0.5377*	−0.5879*	−0.6341*
	CEA	0.0049	0.5651*	0.5164*	0.4718*	0.6763*	0.6124*	−0.4731*	−0.6114*	−0.6634*
	Numerical score	0.201	0.2881*	0.2613*	0.3720*	0.3978*	0.3534*	−0.2705*	−0.3521*	−0.3166*
	NRS	0.209	0.2891*	0.2653*	0.3677*	0.3966*	0.3561*	−0.2638*	−0.3469*	−0.3144*
	Telangiectasis	0.035	0.3637*	0.3919*	0.4758*	0.4953*	0.4646*	−0.3067*	−0.3454*	−0.4146*
≥61	IGA	0.502	0.6982*	0.472	0.301	0.407	0.284	−0.355	−0.466	−0.532
	CEA	0.16	0.8989*	0.6742*	−0.0416	0.629	0.36	−0.423	−0.561	−0.338
	Numerical score	0.467	0.234	0.216	0.389	0.294	0.528	−0.0556	−0.149	−0.36
	NRS	0.467	0.522	0.406	0.389	0.406	0.406	−0.0556	0.0589	−0.0582
	Telangiectasis	0.579	0.6448*	0.572	−0.0197	0.237	0.408	−0.525	−0.478	−0.343

Abbreviations: CEA, clinician erythema assessment; IGA, investigator global assessment; NRS, National Rosacea Society.

* *p* < 0.05.

*Note*: *𝛂* = 0.05.

### 3.3 Correlations between PSA and other parameters

Genome‐wide association study discovered that skin pigmentation genes were associated with rosacea severity.^19^ Spot, UV spot and brown spot represent the pigmentation parameters in VISIA system. Thus, we analyzed the correlations between red area and other VISIA parameters and found that spot, UV spot and brown spot were correlated with red area, with the highest *r* value in brown spot (Table 6). Then, we demonstrated that brown spot showed significant association with IGA (or CEA) (Table 7).

**TABLE 6:** Correlation analysis between other VISIA parameters and red area

	Red area								
	Feature count			Absolute score			Percentile		
Parameters	Front	Left	Right	Front	Left	Right	Front	Left	Right
Spot	0.4273*	0.3229*	0.3229*	0.4010*	0.3469*	0.3469*	0.2720*	0.4200*	0.4200*
Wrinkle	0.1815*	−0.1704*	−0.1704*	0.0614	−0.1530*	−0.1530*	0.0875*	−0.1451*	−0.1451*
Texture	0.1383*	0.0729	0.0729	0.2479*	0.1329*	0.1329*	0.0890*	−0.0910*	−0.0910*
Pore	0.1651*	0.2022*	0.2022*	−0.0441	0.0664	0.0664	−0.0331	0.1602*	0.1602*
UV spot	0.2297*	0.3354*	0.3354*	0.2035*	0.2969*	0.2969*	0.2821*	0.5050*	0.5050*
Brown spot	0.4227*	0.5453*	0.5453*	0.2306*	0.7046*	0.7046*	0.3628*	0.3342*	0.3342*
Porphyrin	0.0845*	0.0332	0.0332	−0.0246	−0.0454	−0.0454	−0.0745	0.065	0.065

Abbreviation: UV, ultraviolet.

* *p* < 0.05.

*Note*: *𝛂* = 0.05.

**TABLE 7:** Correlation analysis between physician's assessment (PSA) and brown spot

	Brown spot								
	Feature count			Absolute score			Percentile		
PSA	Front	Left	Right	Front	Left	Right	Front	Left	Right
IGA	0.1676*	0.2615*	0.2615*	0.0991*	0.2234*	0.2234*	−0.2551*	−0.2205*	−0.2205*
CEA	0.1168*	0.2516*	0.2516*	0.0483	0.2402*	0.2402*	−0.2225*	−0.2193*	−0.2193*
Numerical score	0.0853*	0.0962*	0.0962*	0.1498*	0.0997*	0.0997*	−0.1922*	−0.2233*	−0.2233*
NRS	0.0955*	0.1192*	0.1192*	0.1406*	0.1161*	0.1161*	−0.1973*	−0.2487*	−0.2487*
Telangiectasis	0.0701	0.1898*	0.1898*	0.0647	0.1222*	0.1222*	−0.1610*	−0.0719	−0.0719

Abbreviations: CEA, clinician erythema assessment; IGA, investigator global assessment; NRS, National Rosacea Society.

* *p* < 0.05.

*Note*: *𝛂* = 0.05.

## 4 DISCUSSION

This study revealed that left‐ and right‐side faces of red area in VISIA system were effective in assessing rosacea, especially erythema of rosacea. Red area had the highest correlation with IGA (or CEA) in ETR patients. Among three indexes of red area, absolute score and percentile might be more appropriate than feature count. In contrast, VISIA system in side face could not reflect the severity of rosacea in PhR patients, considering that the manifestations of PhR were mostly phymatous change in the nose instead of erythema in the cheek. To some extent, the feature count of red area in the front face might represent the severity of erythema or telangiectasis in the nose. VISIA system demonstrated no significant correlation with numerical score, NRS or telangiectasia, indicating less effective in PPR and PhR patients in the assessment of rosacea.

VISIA system is composed of a Canon camera with a full‐frame complementary metal–oxide–semiconductor sensor and a quantitative analysis software with RBX technique. VISIA system consists of standard, polarized and UV light sources, identifying spot, wrinkle, texture and pore through standard mode, brown spot and red area through polarized mode, and UV spot and porphyrin through UV mode.^14^ RBX technique is capable of visualizing melanin and hemoglobin via processing red and brown images capture through polarized mode.^15^ Considering the subjective bias in PSA, advanced objective imaging methods would be available to aid in diagnosis.^20^, ^21^ VISIA system, as one of the computer‐aided imaging techniques, has been widely applied in the evaluation of facial pore,^22^ acne,^10^, ^23^ wrinkle,^24^ hyperpigmentation^25^ and rosacea.^26^, ^27^, ^28^, ^29^, ^30^, ^31^, ^32^, ^33^ Here we first demonstrated the correlation between PSA and VISIA system and highlighted the erythema‐directed red area when evaluating rosacea patients. Unfortunately, VISIA system concentrates on the whole image rather topical area, and it could not segment diffuse erythema. Xu combined ImageJ and VISIA system and proposed a simple and precise method to analyze topical erythema.^34^ Besides VISIA system, IPP from Media Cybernetics,^35^ CSKIN from Yanyun Technology^36^ might also be effective in evaluating rosacea. Compared with VISIA system with RGB space, CSKIN used CIELAB color system, which is currently the most widely‐used space, indicating better reliability in assessing rosacea.^36^, ^37^

Despite VISIA system, there are other imaging methods applied in assessing severity of rosacea, including reflectance confocal microscopy (RCM), dermoscopy, capillaroscopy, optical coherence tomography (OCT), ultrasonography and infrared photography.^9^, ^38^, ^39^, ^40^ Among those imaging techniques, RCM and OCT are used for monitoring *Demodex mites* in rosacea, whereas dermoscopy and capillaroscopy were applied for telangiectasis in rosacea. Recently, optoacoustic imaging was regarded as a novel tool to measure high‐resolution hemodynamic changes in microvasculature for monitoring inflammatory skin disorders.^41^ Computer‐assisted diagnosis techniques also broadened our knowledge of noninvasive measurements in rosacea.^42^, ^43^ Although those noninvasive tools seem promising in the assessment of rosacea, adequate and validated clinical trials are needed for further investigation.

## 5 CONCLUSION

In conclusion, VISIA system is an effective noninvasive imaging technique for the assessment of rosacea, particularly in ETR patients. Red area in the left and right faces would be an additional measurement when evaluating the severity of erythema in rosacea patients.

## CONFLICT OF INTEREST

The authors declare that there is no conflict of interest that could be perceived as prejudicing the impartiality of the research reported.

## Data Availability

The data that support the findings of this study are openly available on http://www.medresman.org.cn/uc/sindex.aspx.
